# Gut Healthism: The Penetrating Gaze and Depoliticising Forces of Direct-to-Consumer Microbiome Testing Kits

**DOI:** 10.1111/1467-9566.70111

**Published:** 2025-11-01

**Authors:** Erica Zurawski, Maya Hey

**Affiliations:** 1 Department of Environmental Studies, SUNY College of Environmental Science and Forestry, Syracuse, New York, USA; 2 Centre for the Social Study of Microbes, University of Helsinki, Helsinki, Finland

## Abstract

In recent years, scientific attention towards the gut has interpellated everyday consumers to test and intervene on their gut microbiome in the hopes of improving their overall health. Based on a discursive analysis of direct-to-consumer testing kits, we detail how their rhetoric individualises health interventions in the name of procuring a ‘healthy’ gut microbiome while obscuring the social, communal and environmentally predicated relations that inhere to a kind of health reliant on microbes. Drawing on Robert Crawford’s original conceptualisation, we identify an emergent ‘gut healthism’ amid the tangle of the contemporary microbiome revolution, technopolitical outgrowths of the Human Genome Project, collapsing healthcare infrastructures, and the ills of the modern industrialised food system. Through gut healthism, we argue that the kits enable a hyperfixation on the gut, which becomes mediated by scientific expertise to view and quantify microbes as health markers, whose gaze disembodies guts and depoliticises diet. By examining current moves in gut microbiome products, we also detail the divergences and complications that gut healthism brings to Crawford’s framework, highlighting the problem with solutions such as DTC kits and how they do little to address the grand health challenges of our time.

## 1 Introduction

The gut has recently been celebrated as the ‘center of a biomedical revolution’, a new frontier of science with ‘97% left to discover’ (Nayar 2024; Blaser 2014). This purported revolution has lent itself to myriad claims that fan out from the gut, connecting the status of one’s gut microbiome to the brain and mental health, to more nascent claims that correlate it to heart disease and predispositions of cancers, to even dubious claims linking microbiome health to autism. Long relegated to the status of a digestive organ, the gut now heralds scientific and medical scrutiny, which, in turn, has garnered public attention and promise. Such hype has generated a cascade of science communication books, podcasts and social media channels, epitomising what some have critiqued as ‘microbiomania’ (Eisen 2014) for overselling incipient claims that gut microbiomes are the key to broader health by way of diet.

Although research on the gut microbiome has steadily increased in recent decades, what is acutely novel in recent years is the explosion of direct-to-consumer (DTC) microbiome testing kits, which allow consumers to collect their stool samples and send them to labs for an analysis of their gut microbiome’s composition and concomitant health status. These products are relatively new, emerging as early as 2016 with San Francisco-based uBiome’s SmartGut test, which claims to be the first sequencing-based clinical microbiome screening test. As of this writing, there are now at least 30 companies that sell these DTC kits in the Global North alone, each with an allure of interpreting and translating the health meanings of one’s microbial composition. Although not as large of an industry as the genetic ancestry DTC market, which at its prime was valued at $11.9 billion, the DTC gut microbiome testing industry represents a more stable market given the ability to test and retest compared to the singularity of the genetic testing. As of July 2025, the market was valued at around $1.2 billion, with a projection to reach $3.5 billion by 2033, a significant increase from the 2018 numbers, which valued the market at $637.5 million. Although there is a lacuna of information about the consumer base for these products, they are marketed for everyone and anyone, regardless of health status, a point we develop below. To give some insight into the reach of these products, one company alone claims to have analysed over 600,000 samples with over 200,000 users.

Alongside the scientific studies cashing in on this lucrative microbiome frontier are parallel claims that idealised health has not reached peak optimisation, at least as these DTC companies would have one believe. Instead, individuals must ‘analyze [their] biochemical individuality with mathematical precision to serve up [their] ideal diet’ (DTC Company 1), conveniently enough, through the ease of at-home stool testing kits. The DTC testing kits claim value-added health benefits from their services, including comprehensive health and dietary insights and risk reports, AI-driven apps that function as ‘personal nutrition coaches’ to guide an eater towards healthier and smarter food choices, personalised health plans with precision probiotic and prebiotic supplements to curate one’s own gut health, and even toothpastes and oral lozenges to keep oral microbial activity in check.

As a particularly notable contradiction, these DTC testing kits claim to offer consumers the ability to tell if their microbiomes are ‘healthy’ but do so in the absence of scientific consensus on what exactly a healthy microbiome entails (Van Hul et al. 2024; Najmanová et al. 2022; Bäckhed et al. 2012; Shanahan et al. 2021). A similar mismatch can be seen in the increasing concerns about whether such tests are accurate or, worse, whether the tests ‘could do real harm by convincing a person to delay medical care and substitute supplements for prescription medications’ (Aungst 2024). Scrutinising hope, hype, and contradictory narratives, this article focuses on the social and cultural reproduction of health ideologies in and through these DTC kits.

By drawing attention to the assumptions hidden within commercial discourses of gut health, we ask, what do claims about the gut microbiome and gut health highlight and obscure in relation to ideologies of health? We approach our analysis with disciplinary commitments to sociology, communication studies, science and technology studies, and human geography with a critical eye towards food, eating, technology, and the material-discursive formations that microbes, bodies, and environments take. In what follows, we first situate DTC kits in relation to the promissory nature of gut health and gut microbiome research, followed by a close reading of Robert Crawford’s seminal work on healthism, with focus on the hyper-individualising, moralising, depoliticising, and decontextualising tendencies of healthism’s hegemony. We then apply Crawford’s critique to DTC kits to deconstruct these tendencies within rhetorics and discourses of microbiome wellness while also elucidating the unique tendencies of what we call gut healthism.

Marked by a hyperfocused and penetrating gaze, we offer up the notion of gut healthism as an extension of Crawford’s healthism, which responsibilises individual choice and behaviour as the means to better (gut) health while also assuming that all eating bodies have access to all ‘good’ microbes that can better one’s health. In so doing, gut healthism depoliticises the individual eating body as an unsituated and standalone being while repoliticising a health mandate defined by its microbially situatedness. Ultimately, we argue that gut healthism demonstrates the contemporary relevance and utility of Crawford’s healthism as it simultaneously evokes individualised responsibility to act in accordance with an externalised health ideology while also disembodying the gut from the individual’s body and sociopolitical contexts within which the individual exists.

## 2 Situating Gut Health, Gut Microbiomes and DTC Kits

Understandings of health and illness have gone hand in glove with technological advances. For instance, microscopes and culturing techniques enabled the isolation of pathogens for infectious diseases, and, more recently, research on genetic markers and genomics identified genetic predispositions to disease. As an outgrowth of the U.S. Human Genome Project (1990–2003), the Human Microbiome Project (2007–2016) aimed to map the diverse microbial communities of the human body and their complex roles in health and disease (Turnbaugh et al. 2007). With initial databases released in 2012 and the Human Microbiome Project’s subsequent completion in 2019, a boom in human microbiome research is currently underway at the global scale (Green 2019).

At the vanguard of gut microbiome research are a handful of names—Jeffrey Gordon, Tim Spector, the Sonnenburgs, Jack Gilbert, and Rob Knight—many of whom advise or outright own private companies that have commercialised an aspect of microbiome knowledge, including some of the DTC testing kit companies analysed herein. In addition to general gut microbiome research, these companies offer and market their own scientific advancements and technologies. For example, DTC testing originated with 16s mRNA sequencing that identifies bacteria using short bits of genetic material found on the ribosome, which acts as a barcode of sorts. Nowadays, most companies advertise their use of shotgun metagenomic sequencing, which reads all genomic DNA in a single sample and computationally pieces together a whole code. As one DTC company simplifies, ‘16s is like trying to identify a species of mammal from a single hair’, whereas shotgun sequencing ‘is like identifying a mammal from its hair, bones, teeth, internal organs, and a high-resolution photo of the whole animal’ (DTC Company 2).

The marketisation and capitalisation of the gut microbiome at the ‘academia-industry nexus’ has drawn significant criticism from those who caution about the consequences of a metabolic political economy hallmarked by the commodification and datafication of the human microbiome and the homo-microbial self, of the bioprospecting and assetisation of gut data and the apps and kits therein (Van Wichelen 2023; Kotliar and Grosglik 2023; Raffaetà and Ferrari 2025, 2; Grosglik and Kotliar 2024; Ferrari et al. 2024). As Raffaetà and Ferrari (2025) argue, the gut is not just ‘psychological, cultural, geological, oceanic and meteorological’ but also economic, as it is ‘constituted by political-economic dynamics that enroll it as a valuable agent in a neoliberal framework of health governance’ (Raffaetà and Ferrari 2025, 2), which thus positions the gut ‘as a site where assetization and commodification processes coalesce’ (Raffaetà and Ferrari 2025, 7). As we will elaborate later, the hyperfocus on the genetic information of the gut microbiome demonstrates the penetrating gaze of these DTC microbiome testing kits, exemplifying the degree of reductionism and disembodiment that is unique to gut healthism.

In addition to co-constructing the gut data marketisation and commodification process, these researchers have also been capitalising on the findings from the Human Microbiome Project that have made explicit that the gut microbiome is malleable and thus highly responsive to changes in human behaviour by expounding dietary advice (see, e.g., Nayar 2024; Knight 2015; Sonnenburg and Sonnenburg 2016) even though the jump from mouse models to human models makes the evidential link seem tenuous (Bik 2016, 369; Landecker and Kelty 2019, 63; Raffaetà 2022, 100) and the evidence is self-referential (Bermingham et al. 2024). No study or clinical trial to date has definitively, comprehensively, *and* systematically mapped the complex links between diet and the gut microbiome.^1^

At the same time, social scientists from critical dietetics and nutrition have been increasingly scrutinising the assumptions that get baked into notions of health and modern dietary advice. From critiques of population-scale prescriptivism (Biltekoff 2013; Hite et al. 2010; Broad and Hite 2014) to critiques of singular food facts (Scrinis 2015; Overend 2020), the question of ‘what to eat’ to promote one’s health has highlighted questions of race, class, and geographic location (Hayes-Conroy 2016). Since the height of these critiques in the 2010s, the added overlay of microbiome discoveries has amplified the ableist (Kolářová et al. 2023), colonial (Hobart and Maroney 2019) and gendered/classed (Hey 2020) assumptions that one can simply choose to eat better as a means of controlling one’s gut microbiome and its health outcomes. Stephanie Maroney specifically targets the oeuvre of popular literature penned by microbiome researchers, arguing that ‘microbiome diet books intensify the self-making work of dietary advice, such as individualism, self-control and responsibility for one’s own health’ (Maroney 2020, 42). These critiques coalesce around questions of who can attain hegemonic ideals of health and how, especially when they are increasingly couched in moralised, heteronormative terms.

Similarly, scholars from science and technology studies and the history of science have examined the implications and (dis)continuities of health rhetoric in relation to advances in technology. For instance, microbiome research (along with epigenetics and omics research)^2^ has been increasingly framing health as a metabolic and co-produced phenomenon. Here, ‘food is more than fuel or substrate’ to become a matter of environmental exposure from the inside, due to the porosity and processual character of our alimentary canals (Landecker 2011, 167). Specific to the knowledge-making practices of gut microbiome research, Raffaetà (2022) observes that the data-intensive quantification of microbiomes requires a translation of in vivo microbes into static abstractions in silico so that statistically significant conclusions can be drawn, usually with the help— and errors—of artificial intelligence. The challenges of computationally accounting for microbes in situ require sifting through heterogeneous data, but ‘technology alone is not enough’ because ‘human intervention is always essential for guiding the [AI] system’ (Raffaetà 2022, 100). This means that researchers ‘have an impact not only on the experimental practices of other labs but also on the basic logic of dealing with biological problems’ (Raffaetà 2022, 119). Collectively, these publications attend to the epistemological, methodological, economic and political stakes of health rhetoric when health is, in fact, environmentally predicated and thus context-specific and historically situated.

For gut microbiomes especially, microbial composition is always in flux (or malleable, as scientists describe) in relation to the different microbes one can encounter over time. Beginning with differences observed between vaginal and caesarean births (Shao et al. 2019), gut microbiomes can also differ by the objects one ‘tastes’ and places in one’s mouth in the first 1000 days of life (Finlay and Arrieta 2016), by the presence of household pets (Tun et al. 2017) and, at least for the 36 participants in Wastyk et al.’s (2021) study, by acutely incorporating unsustainable amounts of fermented foods into one’s diet (Wastyk et al. 2021). A key study published in 2018 demonstrated that transgenerational moves from Hmong and Karen communities and refugees resulted in biodiversity loss within 6–9 months of immigrating to the United States, with longer time spent there evincing worse gut compositions, especially in subsequent generations born in the United States (Vangay et al. 2018). An empirical study conducted in 2021 revealed that low socioeconomic status negatively affected the gut microbiome of an entire neighbourhood, which the authors attribute to food insecurity and economic hardship (Zuniga-Chaves et al. 2023; Amato et al. 2021; Gacesa et al. 2022). Microbiomes are biophysical portraits inasmuch as they indicate the social locations from which humans and microbes each can arise. Our microbiome is impacted by environmental and socio-political-economic factors in and through social practices as frequent as eating, as mundane as public transit and as profound as what kinds of medical access (e.g., antibiotics) one can attain or be denied.

Although Widmer (2021) offers a key social science critique on the assetisation rhetoric that DTC kits deploy (e.g., investing in one’s health through DTC kits) and their lasting effects, very few empirical studies demonstrate the scientific efficacy of these testing kits, even if claims have boomed over the years. We also believe that the technology itself has changed since Widmer’s critique (namely, the speed and ease of sequencing), inviting recent calls for the regulation of the industry and more comprehensive analyses on consumer omics and the practices and policies of the DTC genomics testing (Hoffmann et al. 2024; Knoppers et al. 2021). At the time of this writing, there remains little to no consensus on the regulation or proven value of these DTC microbiome and genomics testing kits within clinical practice, largely due to the fact that these kits fall short of delivering actual tailored recommendations and any sort of personalised nutrition (Porcari et al. 2025; Gimpfl et al. 2024). The promissory nature of these kits makes them all the more conducive to healthist claims.

## 3 Revisiting Crawford’s Healthism

Although preoccupations with social practices around health are certainly not new, Robert Crawford denoted a noticeable shift in the importance of, actions surrounding, and ideologies suffusing what he called healthism (Crawford 1980). Unlike prior health practices, Crawford differentiates healthism as a ‘preoccupation with personal health as a primary—often the primary—focus for the definition and achievement of well-being; a goal which is to be attained primarily through the modification of lifestyles, with or without therapeutic help’ (Crawford 1980, 368). Alongside an analysis of the emergence of the middle class in the United States, Crawford argues that the undercurrents of healthism were forged within 1960s American culture and politics, wherein health took on a particularly potent role and focus. From the environmental movement at the time (e.g., in reaction to agrochemical destruction from the so-called Green Revolution) and evidence of the health effects of environmental degradation (e.g., radiation, smog pollution) to the increase of epidemiological studies focusing on chronic disease and findings about carcinogenic foods and products (e.g., tobacco), Crawford paired this with the emerging trends in the 1970s towards self-help and holistic health as seen within the women’s health movement, the organic or natural foods movement, fitness, diet, and jogging culture (Crawford 1980, 368; Crawford 2006). Importantly, these movements demonstrated a notable rejection of the traditional medical establishment and a healthy dose of scepticism towards government intrusiveness into personal freedoms.

Tensions between macro-/societal interventions to health and micro-/individual behaviours came to a head during this time because of deeper and particularly neoliberal political shifts in understandings of personal agency. As Crawford argues, ‘[t]he success of privatized, market solutions to public problems cannot be grasped without a clear understanding of how personal responsibility triumphed over political morality premised on collective responsibility for economic and social well-being’ (Crawford 2006, 409). Thus, Crawford diagnosed a ‘new health consciousness’ of the time, which defined health problems and solutions almost exclusively within the boundaries of personal control (Crawford 2006, 408). Here, personal responsibility was not antithetical to collective responsibility per se, because ‘the personal’ was couched in ‘populist, grassroots, and cooperative efforts as models of alternative ways of providing goods and services’ which were ‘significantly anti-corporate in their sensibilities’ (Guthman 2011, 53). In tandem with the individualising responsibility of healthism came the obligation and, thus, moral valence to health as well. In this sense, to be individually responsible for one’s health helped contribute ‘to the ascendancy of a neoliberal social order’ (Crawford 2006, 409), consistent with the neoliberal political regime (e.g., Reaganomics) of 1970s America.

For the sake of our analysis, we focus on two aspects of Crawford’s healthism—the moralising, individualising responsibility and the depoliticising of health—to explore in detail, as we find them especially poignant for our analysis of an emergent gut healthism.^3^ The first is the overwhelming moral value assigned to the individualised responsibility to adopt behaviour and lifestyle changes to achieve healthfulness. For the healthist individual, the focus, problem, and thus the solution to health rest within a notion of individual behaviour, attitudes and emotions (Crawford 1980, 368). Suffused with an ideology of self-improvement and self-help, healthism’s focus on individual responsibility frames poor health as a result of individual failings, demonstrating the moral valence to health where failure to act, even preventatively, is framed as a kind of deviancy, an un-willingness to be healthy, or an unconscious desire to be sick (Crawford 1980, 379). In this formulation, the omnipresent threat of becoming sick or unhealthy ‘mandates a moral duty’ that, again, rests squarely on the individual to curb unhealthy habits and remain vigilant in healthful upkeep (Crawford 1980, 380).

The second element tangles with the first, where focus on the individual as the locus for health maintenance or change distracts away from macroscale factors in health outcomes such as environmental racism, poverty, or discrimination (Crawford 1980, 378). Rather than situating health within a broader system of complex social structures that shape health and health-related choices, healthism undermines any political or systemic notion of health by ‘reinforcing the tendency towards wholly private, individual solutions’, as if individual motivation and coping are enough (Crawford 1980, 385). Even if some overtures might be made to acknowledge the complex aetiology of disease as originating outside of the bounded individual, such as in the case of environmental pollution or second-hand smoke in the 1960s and 70s, overwhelmingly healthists construct the achievement of health and well-being as personal behaviour (Crawford 1980, 368). The result is a depoliticised and decontextualised conceptualisation of health that ignores or outright denies the structural and systemic macrolevel conditions that shape behaviours, attitudes, emotions, and conditions of health.

In the years since, Crawford’s healthism has been taken up to acknowledge its hegemonic stronghold and diverse applications since the turn of the century (Metzl and Kirkland 2010), as well as to analyse specific ideological undercurrents within the so-called obesity epidemic (Guthman 2011), veganism (Scott 2020), clean eating (McCartney 2016), healthist fermentation (Hey 2020), and food and health marketing discourses (Silchenko and Askegaard 2021), to name a few. Our analysis of DTC microbiome testing kits builds on these analyses to demonstrate how the rhetorics of gut health and its microbial composition reproduce healthism’s individualising and moralising hyperfocus while also absenting the societal contexts that shape individual and community health.

We argue that the contemporary microbiome revolution signals an emergent healthism, which we are calling ‘gut healthism’, based on our analysis of how DTC kits reproduce the underlying logics and ideologies of Crawford’s time. Our analysis shows that gut microbiomes, as an object of analysis, remain indeterminate, unknowable, and thus only partially quantifiable, making it difficult to operationalise at societal levels of dietary or public health guidelines compared to other behaviours such as the smoking or alcoholism that Crawford noted. This microbial gaze renders the scale of the individual body illegible and moot, further disembodying individuals into parts-based, piecemeal formulations of health. In effect, this microbial gaze obscures the broader structural and systemic forces that shape our health, including where and how environmental microbes figure in holistic understandings of health. By situating these DTC gut microbiome testing kits within Julie Guthman’s ‘the problem with solutions’ framework (Guthman 2024), we argue that these kits are narrowly conceived techno-fixes to problems that have been bounded and rendered easily solvable and that do little to understand or address the totality of the problem of improving health.

## 4 Methods

We analysed the discourses and rhetoric of gut health and gut microbial composition articulated by DTC gut microbiome testing companies. We employed a critical discourse analysis because it is a method that is attentive to ‘the way social-power abuse and inequality are enacted, reproduced, legitimated, and resisted by text and talk’ (Van Dijk 2015, 466). With attention to how ‘words are constructed by and construct contextual moments and realities’, a critical discourse analysis was especially useful ‘for understanding language within its “situated network” of meaning, culture, and context’ (Smith 2024, 1309; Gee 1999).

In our analysis, we gathered empirical data from the websites, advertisements and blogs of 27 DTC testing kit companies, which comprise all of the companies we could locate and verify via internet searches, from the period of December 2024 to March 2025. Although many of these companies offer a myriad of tests for oral health, cancer detection, sleep issues, menopause, fertility, thyroid issues, and more, we excluded these other products to focus specifically on the gut microbiome testing kits, given the paper’s focus. Websites, blogs, and advertisements were manually examined through several rounds of recursive analysis, including initial and focused coding, grounded in a guiding effort to understand ‘how findings might extend, challenge, or refine preexisting theories or concepts’ of healthism (Halpin et al. 2025, 3), with particular attention to how these companies leverage ideas and interventions about gut health and how they interpolate individual consumers.

Across the data, we inductively generated three key themes to summarise how gut health and the gut microbiome are related to and possibly extend Crawford’s healthism—discussed below. Although our analysis included 27 total companies and their DTC kits, we cite extensively from six companies based on their prominence, popularity, and market dominance. We acknowledge that these companies are overwhelmingly English speaking and located in the Global North, with most of them located in the United States. Although this represents an important limit to our study, it is nevertheless a technoscientific snapshot in time and place.

## 5 Data and Discussion

### 5.1 Theme 1: The Penetrating Gaze of Gut Healthism

Like any technoscientific company, nearly all of the DTC companies advertise cutting-edge technology to analyse and visualise microbial health, with goals to demystify one’s health, to discover the root cause of disease symptoms, and to elucidate the status of *your* unique microbiome. Companies’ advertisements feature technological capacities for decoding, visualising, and assessing an individual’s gut microbiome. Underlying this technology is the synechdotal promise that accessing the gut microbiome can be a key to visualising one’s overall health. For some, they claim ‘the microbiome influences everything from immune function to mental health’ (DTC Company 5). Others headline the gut microbiome as the ‘chief architect of your health’ (DTC Company 1), claiming that the health of your ‘bugs’ provides insight into your overall health (DTC Company 3). Although we do not take issue with the validity of such statements per se, we baulk at their insistence on the parts-to-whole relationship of gut microbiome-to-overall-health, as it underwrites the need for scientific expertise and equipment to quantify, decipher, and make known the microbial populations rendered invisible to the human eye.

As these companies imagine it, the comprehensive reports resulting from the DTC testing kits offer a unique visualisation of the state of one’s gut. They provide a ‘snapshot of which microbes are present and their proportions in your gut’ (DTC Company 5), identifying ‘down to the strain level, all the bacteria, fungi, viruses and other organisms’ in your stool (DTC Company 6). Identifying microbes here is not only about seeing the seemingly invisible, but also about seeing their particulate effects in situ, individually, not as described on generic self-help websites or in textbook explanations.

At the most basic level, this preoccupation with microbial composition offers an evaluation of ‘good’ versus ‘bad’ gut bacteria inhabiting one’s gut, even though research shows that microbial utility is contextually predicated, not fixed (Walker and Hoyles 2023, 1393; Yong 2016; Walker and Hoyles 2023, 1393). One DTC company claims that its research has supposedly identified the ‘50 “good” species associated with positive health markers and 50 species associated with poorer health markers’ (DTC Company 2), which translates into their DTC testing reports that offer consumers a list of ‘good’ and ‘bad’ bacteria in your gut, with recommendations of specific foods to curate the growth of the good (DTC Company 2). Others up the moral stakes of this binaristic narrative, framing microbial activities as ‘beneficial’ or ‘harmful’, again with the intention of setting the path towards ‘boost[ing] the “beneficial” activities’ (DTC Company 1). This moralistic/binaristic thinking seeps into how the stool sample reports are framed, with customers receiving ‘precision gut health scores’ that ‘score your gut microbiome’ and, in some instances, claim to provide a picture of your ‘gut intelligence’ (DTC Companies 1 and 2). Translating ‘good’ and ‘bad’ microbes into a score frames gut health whereby health becomes a measure of character and self-worth.

We draw extra attention to the work that this visualisation does as an important complication and connection to Crawford’s healthism and the disciplinary power of a Foucauldian clinical gaze, namely, through how ‘clinical discourse is based on the localization of the pathological event within the boundaries of the individual body’ (Turrini 2015, 17; Foucault 1976). Like Crawford’s healthism, gut healthism situates the problem/solution of overall health within the individual; however, unlike Crawford’s healthism, we argue that this gut healthism’s gaze is emblematic for how it so uniquely penetrates into the individual body.

### 5.2 Theme 2: To Test and Retest: Empowering Individuals and a New Moralism

One particular contradiction emerges from the data, as it butts up against Crawford’s healthism. The preoccupation with, hyperfocus on, and resulting visualisation of the gut microbiome and gut health represent a return to and reliance on medical and scientific authority, even if drastically different from more traditional sources and sites of such authority. With many dieticians, specialists, consultants, academics, scientists, and doctors behind the DTC companies or ‘on hand’ for customer consultation, whether through apps, blog posts, or consultations, this finding appears to complicate Crawford’s healthism, which emerged from a strong scepticism of government, a hefty anti-corporate sensibility, and a rejection of medical authority. Even still, the individual empowerment messaging far eclipses the appeals to scientific and medical authority, as the overall purpose of the DTC kits is to ‘empower you to take control of your gut health and wellness’ (DTC Company 5). Although expertise remains exclusive to the technoscience from which these kits emerge, the ongoing services^4^ of these companies claim they empower *individuals* to act in an informed manner.

This theme emerges from nearly all of these DTC kits, with technological advancements advertised as providing unprecedented insights alongside promises to help ‘empower people with the information they need to live a disease-free life’ (DTC Company 1), to ‘take control of your gut health and wellness’ (DTC Company 5), to ‘empower members to make small, highly personalised tweaks’ (DTC Company 4), or to ‘gain a deeper understanding of your gut health and the steps you can take to improve it’ (DTC Company 1). Through personalised readings of metabolic responses to food, breakdowns of microbiome diversity, and health scores, reports from DTC kits claim to curate nutrition and supplementation plans for one’s unique microbiome. Citing the outdated ‘one-size-fits-all’ mantra of modern nutrition and diet, companies promote an approach that caters to the singularity of one’s unique biochemistry derived from AI-driven extrapolations, offering ‘easy-to-digest lessons of food, nutrition, and health … delivered by top scientists … to help you create life-changing nutrition habits’ (DTC Company 2). With personalised week-by-week plans ‘guiding you to make smarter food choices daily’ (DTC Company 2) or personalised food lists that allow you to ‘discover which foods, often perceived as healthy, may not align with your current needs and which foods can actually be beneficial’ (DTC Company 1), gone are the days of ‘relying [on] recommending dangerous fad diets where you’re eliminating entire food groups, or pushing the same generic supplements for every person’ (DTC Company 1). Instead, these companies position their kit as the gateway to actionable changes, such that they assume one can follow through on the promise that ‘small changes to your diet will become habits that can significantly improve your long-term health’ (DTC Company 2). In the words of one company, ‘it’s all about personalization’ (DTC Company 1).

Akin to Crawford’s observations of a new moralism surrounding holistic health and self-help/self-care movements, we also notice similar moralisation in DTC kits with repeated appeals to test and retest as a form of personal upkeep. The allure of retesting is advertised as an opportunity for individuals to not only visualise their gut microbial composition but also visualise ‘which way your gut health is trending over time’ and ‘to understand how you’re moving closer or away from optimal health’ (DTC Company 1). Invoking rhetorics of control and optimisation, the impetus to continually test one’s microbiome, as one company puts it, allows individuals to ‘track your gut health over time and measure the positive and negative changes so that you stay in control’ (DTC Company 1). Building on previous discussions about control over good/bad microbes and of empowerment, consumers are interpellated into an ideology of health and health practices centred around the moral imperative to learn about, balance, improve, control, and fine-tune their gut health—repeatedly—with seemingly no endpoint to testing in sight. One company even asks its customers to ‘imagine a world where illness is optional’ (DTC Company 1), framing a disease-free life as an attainable goal in the illusion that repeated testing can steer individuals away from unhealthy habits and stave off any potential illness.

Through the guise of empowerment and enlightenment via visualising gut microbes, the individualising and self-help sentiment of healthism remains. In this sense, although scientists, doctors, specialists, and even artificial intelligence (AI) are required and advertised to mediate and visualise gut health, they are ancillary to and therefore showcased to support an individual to act better on their own accord. The overall responsibility remains on individuals to seize the opportunity, heed the call of empowerment, and refine their gut for overall health.

Alongside empowerment, the impetus to test and retest is also leveraged by the burgeoning science around microbiomania, with companies claiming that ‘scientific discoveries around the microbiome are moving faster than any one human can compute’ (DTC Company 6). This becomes a rationale for ‘employing AI to help [them] interpret the entire scientific literature on the microbiome and create personal recommendations for your unique composition’ (DTC Company 6). As a commodity, repeated use of these kits promises knowledge ‘of the inside’ from an externalised sample that, over time, allows consumers to visualise and thus know their microbial composition. When paired with how these companies leverage AI as the solution to decoding the millions of data points, the impetus to test and retest is not just about staying abreast of one’s gut health but about calibrating the individualised picture of gut health to the findings from so-called cutting-edge scientific discoveries and developments. This point becomes even more poignant when considered alongside the problem with technosolutionism, which we dive into further detail in our conclusion.

The moralising and individualising responsibility for gut health interpellates every individual to act, not just those concerned about or navigating gut-related issues. Because ‘a gut imbalance can affect your entire body’ (DTC Company 3) and because gut health is framed as the architect for overall health, under the rubric of gut healthism, any health-related concern or symptom can be known and intervened upon through DTC kits, such that to remain uninformed of one’s gut/microbes poses the risk of making ineffective or potentially harmful choices. In this sense, gut healthism extends the danger-consciousness that Crawford noticed within healthism, where seemingly ‘healthy’ foods—not just toxins or unhealthy habits—contain the danger of being potentially harmful. Unlike the threats posed by smoking and drinking during Crawford’s time, gut healthism interpellates all eaters by collapsing every dis-ease and illness under the umbrella of gut health. Framed in this way, it is only through ongoing, repeated, and vigilant gut testing that an individual can identify these potential harms.

### 5.3 Theme 3: The Depoliticising and Decontextualising Tendencies of Gut Healthism

Gut health is understood to be complex, eluding the one-to-one aetiology of diseases such as smoking and lung cancer or alcoholism and fetal alcohol syndrome. From our findings, DTC testing kits provide concessions to the environmental and nonbehavioural factors that impact gut health, predominantly in the form of blog posts that explain complex interactions between microbiomes and air quality (DTC Company 3), emotional stress (DTC Company 3), and urbanisation (DTC Company 1). Or, there is an acknowledgement that ‘the behavior of your gut microbes is shaped by a complex interplay of factors, including diet, lifestyle habits, where you live, stress, and more’, but the conclusion from such an admission underwrites the need to understand real-time effects of microbes in vivo (DTC Company 1), which conveniently warrants the companies’ expertise. Overwhelmingly, these concessions end by answering the question, ‘What can be done?’

Crawford notes in his review across three periods of American culture that the ‘“what is to be done” of securing health is now understood as an intricate and demanding project’ with the underlying implication that ‘health must be achieved’ (Crawford 2006, 402; Lupton 1995). However, as Crawford also notes, the project of securing one’s health takes on new valence as the social, historical, and cultural contexts shift and change. More tangibly, ideologies behind carbohydrates, proteins, fats, cholesterol, salt, and processed sugars have carried differential weights in recent decades, for instance, with some fading to the background or highlighted in the foreground depending on geopolitics, trade negotiations, environmental catastrophes, or the impingement that a looming climate crisis imposes on all three.

And yet, to cut through the din of enumerated challenges, DTC kits neatly tie up the intricacies of health into a simple response. What is to be done? ‘Start with your daily diet’ (DTC Company 3). In individualising the problem of health down to the level of dietary change, we also argue that gut healthism’s penetrating gaze obscures any view of the systemic, structural, and environmental aspects of food politics and the microbes that inhere to food.

This kind of reductionism contradicts how microbiome research has shifted imaginaries about humans in relation to their environments. Nerlich and Hellsten (2009) argue that the shift from HGP to HMP was a systems biology approach, moving away from genes as standalone objects (reductionism) and instead towards understanding humans in context (the term they use is ‘integration’). No longer are humans bounded and sealed off from the world such that their genes express some intrinsic set of traits irrespective of their environments.^5^ Microbiomes epitomised how humans are fluid in-and-with their environments: ‘Traditional genetics saw the body as a container for genes sealed off from the outside world, whereas integrationist systems biology and microbiomics see the body as merging with its environment with genes moving to and fro’ (Nerlich and Hellsten 2009, 33). Although microbes and their genes are understood to be mobile between humans and our shared environments, it appears that foods are not afforded the same imaginary under the rubric of gut healthism.

Although we offer one plausible reason for this lacuna below, we see the ramifications of this dissonance in the way that gut healthism operates. We extend Crawford’s argument that responsibilising individuals absents the environments within which they can act by noting how the penetrating gaze of DTC kits further disembodies and disappears the individual from the contexts and environments within which they undoubtedly exist. Instead, guts become interpellated, and humans are called to act as better caretakers for them. If the problem/solution of health is located squarely in our gut, then we do not have to consider where people live, the stressors they have to cope with, the environmental racisms they endure, the lack of a living wage to afford food, water, or shelter, their (epi)genetic exposure or hereditary diseases, or any other external factor that impacts our overall (gut) health.

This argument is undoubtedly informed by what Raffaetà and Ferrari (2025) have termed the ‘metabolic political economy’, characterised by the ongoing assetisation and commodification of the microbiome, driven in part by the postgenomic condition and the rise of data-driven biology. As others have detailed, this political-economic phenomenon extracts, mines, datafies and commodifies biological material such that it becomes decoupled from its origins and thus from the ‘complex histories, cultural contexts, and biosocial environments’ that shape it (Van Wichelen 2023, 389).

We deepen these conversations around the metabolic political economy by drawing attention to how the discourses within the DTC testing kits depoliticise and decontextualise *both* the people from whom the samples are being collected *and* the food and diets that are prescribed as a result of those samples. One possible explanation for viewing microbes as being environmentally predicated but humans and food consumption not could be in the enduring metaphor of bodies as machines. Associated with the turn of the 19th-century industrialisation (Biltekoff 2013; Overend et al. 2020; Broad and Hite 2014), mechanised understandings of bodies equated food as fuel, with subsequent tweaking of the metaphor to indicate ‘burning fuel’ efficiently or cleanly. With such a longstanding hold on the collective consciousness around food and metabolism, Landecker explains the underbelly of the ‘food as fuel’ metaphor in relation to its depoliticisation:

Food is also the same for everyone, as fuel or substrate; politics lies in how much of what quality of food is available to whom to build laboring, thinking bodies. This model also implies a certain logic of substitution that we still live with: energy bars and drinks are equivalent to meals, and synthetic nutrient supplements may be substituted without effect for their naturally occurring equivalents. One does not need food – one needs energy, and metabolic substrates. This history is […] is both a point of comparison to the new metabolism emerging today, and – thinking very materially – its source.

(Landecker 2011, 173)

Landecker’s argument on metabolic substitutability explains the biological basis for depoliticising food: If all food is fuel, then some fuels can be replaced with other fuels. However, microbiome research intervenes on this rhetoric, precisely because not all foods carry the same microbes or can feed the microbes desired in a so-called healthy gut. We argue that the food-as-fuel rhetoric pervades so strongly that it acts as the predecessor—or missing link, perhaps—between the rationales that ‘everyone needs to better *food* [to better fuel one’s machine-like metabolism]’ and ‘everyone needs better *microbes* [so that those microbes can better process the fuel/substrate that we call food].’ In this formulation, gut healthism’s penetrating gaze not only disembodies the individual but also mechanises and technologises it, rendering the individual as a knowable, hackable, fixable, tweakable, and optimisable machine. In turn, the substitutability of fuel omits the geopolitical, deeply colonial, and highly extractivist matters that inhere to food.

Gut healthism obfuscates these two threads—the shift from reductionism to systems biology that mirrored the shift from genomes to microbes, as well as the obdurate metaphor of food as fuel that renders foods as interchangeable and thus apolitical. However, these threads tangle differently in the contemporary moment due to the immense hype and promissory nature of gut health. Food is meant to feed certain microbes and not others, DTC testing kits are meant to give periodic snapshots of this feeding efficacy, and yet, the food-as-fuel rhetoric lumps all food into a kind of sameness that only algorithms, apps, on-staff experts, and personalised recommendations can parse and formulate into a gut intervention. The situated, contextual, and socially contingent dimensions of food are chucked out of frame with gut healthism’s penetrating gaze, even though the systems biology approach to microbiome research continues to view the dynamicity and fluidity of microbes as being key to the latest aspirations for (gut) health.

Socioeconomic factors are also external to an individual and exert their force on scales grander than simply one’s choice of which foods to eat for one’s gut health. Gut healthism, however, removes from view the broader structural and systemic causes of health inequities. The effect, as Crawford notes of healthism generally, ‘reinforces the illusion that individual coping is enough’ and ‘reinforces the tendency towards wholly private, individualised solutions’ as it ‘undermines a political conception of the health problem’ (Crawford 1980, 385). Some scholars have already identified the political apex of modern gut health, be it strategic removal of fibres—crucial fodder for gut microbes—from the modern industrialised food system (Maroney 2020; DuPuis 2016), be it normative understandings of able-bodied guts and the narrow definition of gut health that follows an imperative of normalcy (Dryden 2023), or be it unmitigated growth and a life of excess courtesy of capitalism (Richardson 2024). Yet, the hegemonic sociopolitical mindset keeps gut health a personal problem to withstand or contend with in the shadows of privacy.

## 6 Conclusion

In the absence of consensus around definitions of gut health, DTC testing kits underwrite the continual optimising of one’s microbiome as a uniquely *unquantifiable*, Sisyphean task, with emphasis on testing and retesting to empower oneself towards good moral standing. Although other health markers can be monitored with smoking, drinking, and eating so that what and how much one consumes can be adapted to attain an idealised health, microbially-inflected gut health remains evasive in its ever-shifting nature and, thus, only actionable by those who are compelled and can afford to continually retest. In line with the findings of Widmer (2021), DTC kits financialise health as an investment, but even more than this, our findings point to a key contradiction: The malleability of the gut microbiome is both the promise and the necessity of the DTC kits. The malleability of the gut microbiome means that anyone can change their microbiome. However, that requires periodic and repeated snapshots of said microbiome, through DTC testing kits, of course. In addition to being a pricey endeavour, the notion that such a simple solution exists to improve one’s microbiome is moot because microbiomes can be affected by factors beyond dietary intake. These factors exceed the individual, implicating complex challenges at the macro level of collective, communal, and environmental struggles. In this sense, the insistence on changing personal behaviour points to a political conundrum: What exactly are these kits intended to solve and for whom?

Although underwritten by and suffused with Crawfordian healthism logics, gut healthism could have reimagined a different microbial politics. The hyperfocus on the gut as a means to visualise the microbiome *could* signal an opportunity to politicise gut health (and health writ large) as a more comprehensive understanding of (gut) health as simultaneously political, social, economic, environmental and communal. Alternatively, calling attention to the bio- and geo-diversity of microbes *could* offer a more embodied, grounded, and politicised understanding of (gut) health that remains attentive to the embodied politics and lived experiences that shape individual, collective, and planetary health. If, indeed, gut health is predicated on the right microbes and the right food for gut microbes, we *could have been* seeing interventions that address the root social causes of food access, food quality, food affordability, and food production, to start.

However, in practice, gut healthism’s penetrating gaze disembodies the individual and depoliticises gut health to a matter of mere choice. Instead, the kits remain a solutionist techno-fix that is little more than a ‘product of our present condition, not a response to it’ (Guthman 2024, 5). We draw attention to this tension between the potential for (re)politicisation of gut *health* and the resultant depoliticisation of gut *healthism* to add empirical texture to the conjunctural politics that differentially shape contemporary discourses on health ideologies. Our attempts at mobilising gut healthism are intended to signal important divergences and complications of Crawford’s original conceptualisations.

Like Crawford’s healthism, gut healthism’s own emergence is undeniably shaped by the politico-economic shifts of the 21st century and the burgeoning industries and technological advancements within and beyond health and medicine. Alongside the growing popularisation of gut health—evinced through documentaries, children’s books, popular magazine articles, and self-proclaimed gut health influencers—technological and scientific advancements capitalise on the logics of gut healthism. We see this groundswell as a continuation of Crawford’s healthism, where the privatisation and ongoing dismantling of American healthcare systems have entrenched the neoliberal ideologies of healthism. However, unique to gut healthism is how these individualising and neoliberal ideologies are likewise sedimented with the techno-solutionism unique to our time.

Gut healthism’s reliance on technological innovation, data prospecting, and digitalisation requires another analytical tool-kit to make sense of the role that innovation plays in coconstructing gut healthism. Thus, in this concluding section, we turn to Julie Guthman’s recent work on the problem with solutions in her book of the same name (Guthman 2024) to argue that the solutions offered through gut healthism—namely, the DTC testing kits—are better understood as an empty solution, not a response, to the problem of gut health. Under Guthman’s analytic, a solution is hallmarked by its ‘understanding [of] a problem only in solvable terms’, which ‘begins to set limits on the range of possibility of addressing the manifold crises we face in the world’ (Guthman 2024, xiii). The problem with solutions is that they epitomise the ‘finite, narrowly conceived fixes to problems that themselves have been bounded and rendered solvable’ (Guthman 2024, xiii).

In addition to limiting the frame of what exactly needs addressing and how, solutionism puts ‘the cart of the solution in front of the horse of the problem’ (Guthman 2024, 9), where solutions, especially in the form of technological advancements, are developed ‘in advance of or in disregard of problems’ (Guthman 2024, 6). As Guthman argues, ‘many current-day technologies … don’t solve anything of great importance … and actually reproduce many of the conditions and structures innovators putatively aim to disrupt’ (Guthman 2024, 4). Similarly, these technological solutions ‘don’t contend with the inextricable social roots of many of the world’s food and agriculture problems’ (Guthman 2024, 4). Instead, the failure of solutions lies in their attempt to ‘apply technology towards complex problems that demand *political* solutions; under-research or neglect the underlying problem to sidestep what might best address it; and prioritize the ideas, values, needs, satisfaction, and expertise of those delivering the solution rather than the intended beneficiaries’ (Guthman 2024, 15–16, emphasis added).

In a similar vein, although the DTC testing kits offer the allure of the ability to peek into the mysterious inner workings of our gut, these kits and the appeals to gut healthism are not ‘up to the task of changing what needs changing’ (Guthman 2024, 5). As we have detailed, DTC testing kits detract attention away from the totality of the problem of (gut) health and fail to recognise the root social, environmental, economic, and political causes of (gut) health issues. Although the DTC companies claim to be an arbiter for transformative health outcomes, in reality, these kits do little more than ‘empower’ customers with information about their microbiome, ‘a textbook example of solutionism’ (Guthman 2024, 124). Such an impulse towards more precision, more personalisation, and more information begs the question of whether that precision is even needed or wanted in the first place. In short, when seen alongside health-adjacent demands for living wages, better and more robust healthcare systems, solutions to or mere acknowledgement of climate change, and more just and equitable food systems, it seems nobody really asked for this. Even more to the point, the problem with gut healthism is not just in the technosolutionism by way of the DTC testing kits but also in the way that the DTC gut testing kits produce and reproduce age-old logics and ideologies of healthism, further sedimenting them within individual bodies through the penetrating microbial gaze. In this sense, the synergy between the narrow vision afforded by the problem with solutions and the hyperfocus on the gut does little, if anything, to address the trenchant and interwoven social, political, and environmental health issues of today.

## Data Availability

The authors have nothing to report.

## Footnotes

1 We acknowledge that the term ‘microbiome’ refers to the cumulative *genomic* contents of a microbial sample and not the collection of microbial *species* therein. Nevertheless, we use the latter sense of the term as it is used in the promotional materials of our DTC kits under study. Similarly, we recognise that microbes do not inhere solely to the gut, but our paper nevertheless takes up gut microbiomes as the focus of our inquiry.

2 Omics research refers to the study of identifying and quantifying their cellular functions based on the various constituents of a cell (e.g., studying genes, proteins and metabolites as genomics, proteomics and metabolomics, respectively). Microbiology has particularly benefited from metagenomics or the (meta) study of environmental DNA, which often includes the genetic traces of microbes. Although previous lab methods have had to culture live microbes (of which only a small fraction can survive under laboratory settings), metagenomics looks at all microbes who were once there, thereby giving an environmental snapshot of microbial communities heretofore unculturable. See Chapter 4, ‘Studying Microbes: Wet Biology and Dry Biology’, in Raffaetà (2022) for further discussion.

3 Crawford’s more comprehensive framework of healthism includes a preoccupation with personal health and well-being; a hyperfocus on individual responsibility to achieve said health specifically through behaviour and lifestyle modifications; a rearrangement of power away from physicians and professional medicine to instead put the locus of control within the individual; a twin duty of upholding health as the pinnacle moral good and ascribing moral failing to those suffering from poor health; and a depoliticisation of the understandings of health, which divorced health from the societal, systemic and structural conditions that undeniably shape the conditions for health (Crawford 1980).

4 The service aspect of these companies serves a crucial function, because early genetic testing kits (like 23andMe) could not find a financial means of sustaining their business with test results presenting static data.

5 The field of epigenetics also quashed this assumption.
